# Development of a Group Psychotherapy for Combat Veterans With Moral Injury: Protocol for a User-Centered Study

**DOI:** 10.2196/87756

**Published:** 2026-05-27

**Authors:** Sheila O'Brien, Marianne Goodman, Brett Litz, Ghislaine Boulanger, Erin Finley

**Affiliations:** 1 VISN17 Center of Excellence for Research on Returning War Veterans, 4800 Memorial Drive, 151C, Waco, TX, 76711, United States, 1 2544006742; 2 Bronx Mental Illness Research Education and Clinical Center (MIRECC), James J. Peters VA Medical Center, Bronx, NY, United States; 3 Department of Psychiatry, Boston University School of Medicine, Boston, MA, United States; 4 Psychology Department, Boston University, Boston, MA, United States; 5 Postdoctoral Program in Psychotherapy and Psychoanalysis, New York University, New York, NY, United States; 6 VA Greater Los Angeles Healthcare System, Los Angeles, CA, United States

**Keywords:** moral injury, veteran, group psychotherapy, treatment development, user-centered design, psychodynamic

## Abstract

**Background:**

Approximately 25% of combat veterans with posttraumatic stress disorder (PTSD) seek treatment for traumas involving potentially morally injurious events (PMIEs), which involve acts of commission, omission, or betrayal that deeply transgress one’s sense of right and wrong. The sequelae of exposure to PMIEs, called “moral injury,” are associated with functional and psychiatric impairment and disrupt veterans’ sense of identity and meaning, ability to connect with and trust others, and engender disturbing guilt, shame, rage, and disgust. Currently, no first-line treatments directly address moral injury, and evidence-based treatments for PTSD may be limited because they were derived from civilian contexts, poorly fit the war zone context, and do not allow veterans to discuss the details of the PMIEs with other veterans.

**Objective:**

We propose a depth-oriented group psychotherapy for morally injured US combat veterans. Its development is guided by user-centered design principles.

**Method:**

This research plan will use user-centered design methods that continuously gather user experiences during treatment development, with the goal of increased effectiveness and usability. Aim 1 is to discover user needs and preferences as well as treatment-engagement barriers and facilitators from the perspectives of PMIE-impacted veterans and the US Department of Veterans Affairs (VA) trauma clinicians (ie, mental health providers and chaplains) using semistructured qualitative interviews. Aim 2 is to design a treatment manual and refine it using feedback from veterans, VA clinicians and chaplains, and an expert clinical advisory board. Aim 3 is to conduct 2 rapid prototyping open trials (ie, tangibly testing treatment approaches using a prototype manual) with PMIE-impacted veterans (N=12) and iteratively revise the manual based on veteran, provider, and clinical expert panel feedback. We hypothesize that the treatment manual will meet usability, feasibility, learnability, and acceptability criteria.

**Results:**

This study was funded with a start date of November 2021. Participant recruitment for Aim 3 pilot trials began in October 2023 and ended in January 2025. We anticipate study data collection and primary data analysis to be completed in January and April 2026, respectively.

**Conclusions:**

This project aims to develop and pilot test a treatment manual for a depth-oriented group psychotherapy for moral injury. We anticipate that the manual will meet predetermined usability, feasibility, and acceptability criteria because we used user-centered design methods; if it does not, we are well-poised to revise the manual based on user feedback. Should primary outcomes be met, the next step will be to design and execute a parallel-group randomized controlled trial. We additionally plan to disseminate learnings about combat veteran and VA clinician needs, preferences, and barriers regarding moral injury group psychotherapy, which may inform treatment development efforts beyond this project and study team.

## Introduction

### Background and Significance

War zones are distinguished by the potential for engaging in and becoming traumatized by actions taken or not taken that seem to involve moral violations and to result in significant suffering, harm, or death. War zones are rife with moral complexity, and some combat events are disturbing because they involve acting or being treated in morally transgressive ways rather than, or in addition to, surviving life-threatening events; these have been labeled potentially morally injurious events (PMIEs) to distinguish them from the prototypical life threat–based traumas defined in “Criterion A” of the posttraumatic stress disorder (PTSD) diagnosis [1]. Examples of PMIEs include killing either combatants or noncombatants, participating in or failing to prevent excessive violence, or being unable to intervene on behalf of others’ suffering. The prevalence of PMIEs likely varies by branch, unit, military occupational specialty, and era. However, approximately 40%‐60% of Operation Iraqi Freedom (OIF) and Operation Enduring Freedom (OEF) combat infantry soldiers and marines reported killing enemy combatants, and approximately 10%‐30% reported killing noncombatants; a minority of veterans report engaging in or witnessing excessive violence or atrocities [2-4].

PMIEs must be understood in the war zone context in which combat events may be simultaneously transgressive and life-threatening. The likelihood of reporting high-magnitude PMIEs such as killing or engaging in excessive violence is strongly associated with the intensity of combat [5], and more so than known trauma risk factors such as adverse childhood events [6]. Vietnam and OIF and OEF veterans describe how being overcome by grief, anger, and a desire for retribution for lost unit members had motivated their excessive violence [7,8]. Similarly, deployed service members were more likely to report mistreating noncombatants if they also reported that a member of their unit had become a casualty, if they had handled dead bodies or human remains, or if they were angry [9]. Thus, it is important to recognize that PMIEs may be considered an expected part of participating in intense combat and not due to personal characteristics of the veteran. Although combat exposure appears to be associated with reporting PMIEs, not all combat veterans experience PMIEs. Relatedly, PMIEs do not necessarily entail lasting harm, but there is accumulating evidence that PMIEs have a higher conditional probability of resulting in lasting harm.

In general, PMIEs in combat are associated with worse behavioral and functional outcomes, over and above the known impact of general combat exposures [1,10]. Putative PMIEs, like killing and exposure to or participating in excessive violence, are consistently associated with the increased likelihood of mental disorders like more serious PTSD, depression, substance abuse, and functional impairment, even after accounting for the known contribution of life-threatening combat experiences. Reporting PMIEs increases the liability of known risk factors for suicide, such as PTSD and depression. Thus, for some veterans, it seems there is something incrementally toxic about actions taken in combat that resulted in actual or perceived harm to another person.

**Figure 1.**
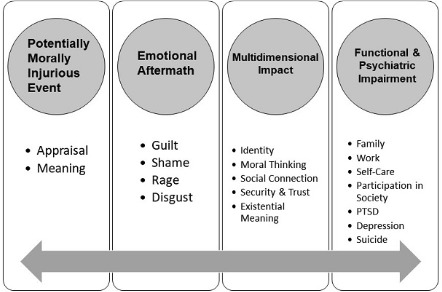
Model depicting the proposed bidirectional impact of potentially morally injurious events. PTSD: posttraumatic stress disorder.

PMIEs are proposed to result in a complex trauma syndrome characterized by chronic reexperiencing, avoidance and numbing, entrenched guilt, shame, rage and despair, isolation and severing of social ties, self-harm and, in extremis, suicide; this putative syndrome has been labeled moral injury [11]. In distinction to life threat–based combat trauma; PMIEs that involved actions the service member or veteran took were associated with more trauma-related guilt, reexperiencing symptoms, negative thoughts about oneself, self-blame, and sadness, whereas PMIEs that involved witnessing others’ transgressions were associated with more betrayal, humiliation, and engaging in physically assaultive events [5]. See Figure 1 for a model of PMIE-related impact.

However, there is no consensus around the boundaries of the moral injury syndrome or how it is distinguished from PTSD. Major questions about the construct validity of moral injury remain unanswered. There is no paradigmatic way of defining moral injury as an outcome, and causal frameworks for moral injury are largely untested and speculative [10]. Litz et al [11] proposed the first conceptual model of moral injury; many variations of this definition have been circulated, and there is little guidance on how to synthesize these varied definitions. Because moral injury is typically conceptualized as a complex trauma syndrome, it is unknown whether the idea of moral injury has incremental clinical or explanatory validity over established psychiatric diagnoses such as PTSD and depression. Relatedly, there is no field-wide agreement about whether moral injury is a syndrome, a diagnosis, or a subtype of PTSD. Attempts to distinguish moral injury from PTSD have generally relied on straw-man notions of PTSD and moral injury or have used methodologically flawed empirical strategies to disaggregate them [12]. In sum, the notion of moral injury is like a bead of mercury; it appears stable and unitary but when pressed, skitters apart.

This project will focus on developing a treatment manual that will be designed to target the lasting and varied multidimensional impact of PMIEs, rather than targeting a vague notion of the moral injury concept. Preliminary studies supporting this project have used qualitative interview methods with service members and veterans to generate reliable domains of PMIE-related impact. These domains include alterations in self-perception and identity; alterations in one’s moral thinking; alterations in feelings of social connectedness and changes in social behavior; alterations in expectations regarding security and trust; self-harming and self-sabotaging behavior; profound emotional aftermath such as anger, guilt, disgust, and shame; and alterations in beliefs about life meaning and purpose [13]. These data reflect service members’ and veterans’ phenomenological perspectives of the impact of PMIEs and will inform treatment development efforts. Given that moral injury is a complex trauma syndrome, we will focus treatment on PMIE-related impacts on functional impairment and quality of life.

Existing PTSD treatments may inadequately address the morally injurious aspects of combat trauma because they were developed for civilian trauma contexts. These treatments were developed for single-incident victimization-based trauma exposures such as adult sexual assault or surviving a terrorist attack. The US Department of Veterans Affairs’ (VA’s) trauma-focused treatments of choice typically use one of two change strategies: (1) identifying irrational or inappropriate trauma-related attributions, such as erroneously assuming responsibility for one’s trauma, challenging these cognitive distortions, and replacing them with purportedly more accurate thoughts (cognitive processing therapy [CPT]); or (2) repeated imaginal or in vivo exposure to trauma reminders with the aim of extinguishing or habituating to overgeneralized trauma-related stress reactions (prolonged exposure [PE]). See Table 1 for a description of therapy activities. The idea of PMIEs may help explain why evidence-based PTSD treatments developed in civilian trauma contexts that are effective in those contexts (CPT and PE) are significantly less effective in service member and veteran populations [14]. For example, approximately 70% of service members and veterans who complete PE or CPT do not report clinically significant symptom change or lose their PTSD diagnosis [15-17]. These evidence-based approaches may have limited applicability to war zone PMIEs because of the following reasons:

1. These approaches require patients to select one “worst and presently most distressing” trauma to treat in therapy. Adult civilian traumas are presumed to be a single incident in an otherwise non–life-threatening context; however, combat deployment is a chronically dangerous endeavor, and veterans confront many life-threatening situations as occupational hazards. Thus, we suspect these approaches do not reflect the reality of veterans’ war zone experiences because they presume that combat traumas, including PMIEs, are isolated and rare events and thus do not allow for PMIE-related alterations in self-perception and identity or moral thinking.
2. Cognitive-behavioral trauma therapies such as CPT involve identifying faulty trauma-related assumptions of blame and ameliorating those assumptions. Because evidence-based PTSD therapies were developed to treat life threat–based or victimization-based experiences, trauma-related guilt and shame are presumed irrational and indications of internalized victim-blaming. However, in the war zone context, veterans’ beliefs about their responsibility for the consequences of their actions may be accurate. Importantly, it is contrary to military training, cultural values, and norms to suggest that blame, responsibility, or culpability is an illusory construction. Military doctrine instructs that service members are responsible for even unintended secondary- and third-degree consequences of their actions; this needs to be calculated into trauma processing. Additionally, qualitative research with veterans has found that some veterans feel guilt not just because of what they did, but also because at the time they enjoyed carrying out violent acts [8]. Guilt, shame, and anger may not be an irrational response to PMIEs but an appropriate one and a sign of an intact conscience and meaning-making system. In the principal investigator (PI; SO)’s prior ad hoc therapy group, veterans spurned the idea of losing their sense of blame and responsibility. We may speculate that veterans’ sense of blame and responsibility (and associated guilt and shame) helped them rebuild a sense of order and justice out of war zone chaos; they intellectually recognized they were not solely to blame, but cognitive appeals to reason did not meet their strong affective sense of culpability.
3. Additionally, there is little evidence or theoretical support to suggest that guilt, shame, sadness, or rage abate following repeated exposure to avoided trauma-related stimuli (as in PE).
4. PE and CPT are not well-suited for group treatment, which is a cost-saving way to treat veterans and may provide veterans opportunity for exposure to corrective experiences stemming from discussion and feedback from other veterans who share their experiences. There is a group CPT protocol; however, it does not include group-based trauma processing and proscribes direct disclosure of combat traumas. In sum, the theoretical and cultural relevance of existing trauma-focused treatments for combat-related PMIEs may be curtailed.

**Table 1.** Brief description of existing posttraumatic stress disorder (PTSD) and potentially morally injurious event (PMIE)–focused treatments.

Treatment	Format	Target	Change agents	PMIE^a^ disclosed	Goal
PE^b^	Individual	Single trauma	Exposure and extinction of trauma-related stimuli	Yes	Reduce PTSD^c^ symptoms
CPT^d^	Individual or group	Single trauma	Cognitive restructuring	No	Reduce PTSD symptoms
ACT^e^ for MI^f^	Individual or group	Moral pain	Behavioral activation, acceptance of moral pain	No	Increase functioning
Impact of killing	Individual; yoked to PE or CPT	Single killing trauma	Cognitive restructuring	Yes	Reduce PTSD and improve functioning
Adaptive disclosure	Individual	Single PMIE	Disclosure and imaginal dialogue	Yes	Reduce PTSD symptoms and improve functioning

a PMIE: potentially morally injurious event.

b PE: prolonged exposure.

c PTSD: posttraumatic stress disorder.

d CPT: cognitive processing therapy.

e ACT: acceptance and commitment therapy.

f MI: moral injury.

Interventions that purport to directly target PMIEs are being developed and tested in the VA. These include acceptance and commitment therapy for moral injury (ACT-MI) [18], adaptive disclosure (AD) [19], and the impact of killing (IOK) intervention [20]. See Table 1 for a description of therapy activities. However, nascent PMIE-focused therapies may be limited because (1) there is no consensus definition of moral injury or gold-standard outcome measures; (2) IOK and AD are constrained in the type or number of PMIEs treated; (3) neither ACT-MI, IOK, nor AD are designed for PMIE processing in a group context; (4) ACT-MI, IOK, and AD have limited efficacy evidence.

There is no consensus definition of moral injury as an outcome and no gold-standard treatment outcome measure. Not surprisingly, different therapeutic strategies that purport to treat PMIEs often use divergent definitions of moral injury, and, to date, any formal approach is limited by a lack of a treatment-valid outcome measure of moral injury. For example, ACT-MI therapy narrowly formulates moral injury as maladaptive attempts to manage moral pain following events that violate one’s morals, including others’ actions that victimized the veteran [18]. These strategies used to manage distress and suffering are also core causal constructs and targets in ACT, regardless of the condition being treated. So, the narrow definition of moral injury used by ACT-MI appears to be primarily the result of the theoretical framework of a treatment, rather than a well-rounded way of defining moral injury. A problem with this narrow definition is that it assumes that meta-cognitive strategies that people use to manage moral emotions are in every case a result of strategic attempts at controlling the experience. It assumes that if these meta-cognitive strategies are reduced, moral emotions will abate, which are unaddressed empirical questions. In addition, conceptually the narrow targeting of ways of coping with moral emotions leaves out a variety of other well-described core features of moral injury (being treated as or perceiving the self as an “other,” social withdrawal, etc). AD is designed in part to treat moral injury but defines moral injury by virtue of the traumatic insult (the worst and presently most distressing event) and measures the efficacy of the approach in functioning and PTSD terms.

PMIE-focused therapies target a limited set of PMIEs. IOK and AD target PTSD and functional impairment secondary to war-related PMIEs. The IOK intervention intentionally limits its focus to PTSD symptoms associated with killing in combat and is meant to be adjunctive to PE or CPT. AD does not limit the therapy focus to a single type of PMIE; however, AD requires that a veteran choose one military event as presently most distressing and haunting at the time of this writing, and the aftermath of this event is the initial focus of treatment. Veterans must make a forced choice of a single “worst” combat event to focus on in therapy, which may be frustrating and invalidating for some veterans.

No PMIE-focused therapies or any recommended PTSD treatments support veterans’ disclosing and processing their PMIEs with each other facilitated by a group leader. Existing PMIE-focused therapies (ie, ACT-MI, AD, and IOK) do not provide social contexts that allow veterans to share and process their PMIEs and war zone experiences with other veterans. Additionally, the VA/Department of Defense (DoD) PTSD Clinical Practice Guidelines do not recommend any group treatments wherein veterans can disclose and process their traumas with each other [21]. VA clinicians have likely developed local group therapies to provide these therapeutic experiences, but there are no treatment manuals or clinical guidelines to support these practices. In fact, there is surprisingly little research on group treatments for PTSD, either in or outside the VA, despite its frequent use and interest to veterans [22]. For example, in a rigorous national survey of Iraq and Afghanistan veterans’ treatment interests, nearly 40% of veterans expressed an interest in group therapy [23]. Veterans in the PI’s informal ad hoc psychotherapy group informally reported that the group was one of the first opportunities to speak about their combat traumas in therapy with other veterans. We suspect that by not providing veterans with treatment groups in which they can share their PMIEs, veterans may not have any opportunity, in or outside therapy, to share their most troubling experiences with the only other people who they perceive will “get it.” Veterans informally reported that they have never told anyone about their experiences because they do not talk about their PMIEs in other therapy groups, and informal or social reunions with comrades or former units are not spaces to share disturbing and haunting memories. As they note, veterans mostly hang out with other veterans, but they avoid talking about their PMIEs. The VA could be unintentionally communicating to both veterans and providers that these experiences cannot be put into words or should not be shared. Without training and support, providers may feel unprepared to directly discuss these events [24].

Because of the potential limitations of existing PMIE- and PTSD-focused treatments, we believe that veterans will benefit from a group treatment that provides an opportunity for disclosing PMIEs with other veterans, processing the strong affects associated with these events, accepting the reality of being changed by and the universal human impact of PMIEs, and directly addressing alterations in meaning and purpose.

We will develop a usable, feasible, learnable, and effective manual guided by best practices from implementation science. Treatment design (ie, form and function) is a central but often underappreciated determinant of treatment effectiveness. Speculatively, design issues may account for why therapies that are efficacious in tightly controlled clinical trial settings falter when applied in real-world settings; for instance, manuals may be difficult to learn or implement or have a poor fit within a delivery context. We plan to use 2 cutting-edge treatment development strategies from implementation science to develop our therapy manual: the user-centered design framework and the stage model for treatment manual development.

**Figure 2.**
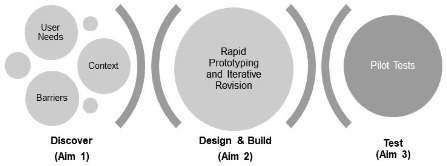
The Discover, Design and Built, and Test (DDBT) model forms the basis of this Career Development Award-2 research plan.

The user-centered design framework organizes the process of developing evidence-based treatments into 4 stages, that is, Discover, Design and Build, and Test (DDBT). See Figure 2 for the DDBT model, based on Lyon et al [25]. The user-centered design framework provides functional guidance for involving end users and stakeholders in every stage of treatment design, from initial idea development to final implementation strategies. In practice, the application of these methods substantially increases the likelihood of creating a treatment that can be rapidly and efficiently learned and used by providers because it accommodates feedback from clinicians and patients. Because user-centered design intentionally canvasses both patients and providers, it results in a treatment that is acceptable and feasible for both types of users and addresses clinical system and setting needs and potential constraints.

The goal of a treatment manual is to provide a description of a therapy and instructions for therapists on implementing it. However, the manual needs to include different types of information and levels of detail depending on the stage of treatment development. Therapists in a pilot trial have different needs than a therapist in ongoing regular clinical practice. The stage model for treatment manual development outlines what a treatment manual should include for a pilot trial (vs an efficacy trial or ongoing clinical care) [26]. Treatment manuals for pilot trials should include preliminary specification of techniques, goals, format, and active ingredients; these manuals are written in such a way that they can be iteratively revised and refined following the pilot test and future efficacy or effectiveness trials. The stage model for treatment manual development specifies seven chapters that a pilot test manual should include (1) an overview, description, and rationale of the treatment; (2) conception of the disorder or problem; (3) treatment goals; (4) contrast to other approaches; (5) specification of defining interventions; (6) session content; and (7) general format.

As part of her clinical responsibilities, the PI informally developed a group psychotherapy to redress the limitations of existing treatments. This group was based on relational dynamic theory. The Relational School of Psychoanalysis grew out of a series of books and papers by Mitchell [27] first published in the 1980s. The Relational School emphasizes the role of relationships with others, real and imagined, in constructing mental life including mental disorders and distress. Working with Vietnam veterans and survivors of other traumatic events in adult life, GB (coinvestigator) developed a theory of adult-onset trauma embedded within the relational tradition. Under the circumstances, this work is seen as applicable in the PMIE context [28].

Adult-onset trauma is viewed as a result of catastrophic disruptions to the familiar senses of the self; for example, the senses of time and agency become dysregulated, but in particular the affective self is flooded with feelings such as fear, guilt, and shame that disrupt self-perception and destroy a previous experience of a cohesive and continuous identity from pre- to posttrauma. Equally significant, the ability to feel connected to others and the sense of relatedness are undermined. A principle of relational dynamic therapy is that specific constellations of memories, emotions, situations, and relationships hold key meanings about earlier events, in this instance, about combat. We suggest this conceptualization aligns with service members’ and veterans’ reports of the impact of PMIEs [13].

The goal of relational dynamic trauma therapy is to help veterans identify connections between their present symptoms and their experiences in combat and their PMIEs, their present life stressors and relationships, and the historical factors that carry person-specific meaning to their trauma or PMIEs. These explorations take place in the presence of attuned and sympathetic others, who can resonate with the experience and the affect being expressed. Symptom reduction occurs through increasing the patient’s capacity to consciously reflect on their experiences and develop an integrated self-awareness of the various factors that affect their mental states. The result is greater self-reflection, less avoidance, and greater adaptive incorporation of life experiences and their aftermath and meanings into one’s inner world. The mechanisms of change in dynamic therapy include identifying and helping patients to understand the emotional and behavioral antecedents to their symptoms; developing an awareness and exploring the meaning of psychological conflicts around strong affects such as aggression, dependency, vulnerability, and caring that are associated with their PMIEs; identifying the unconscious (automatic) and nonverbal behaviors with which veterans manage their conflicts; and proposing hypotheses about the meaning that relates these aspects of the veteran’s experiences of themselves. Over and above all, this work is undertaken in the presence of others who can validate without judging the individual experiences being reviewed; this is of paramount importance in relational dynamic treatment with patients who have been traumatized [29].

Broadly speaking, dynamic therapies effectively target psychosocial and interpersonal functioning, independent of psychiatric labels [30], including depression, grief, and anxiety [31,32]. Although few controlled trials have tested the impact of dynamic therapies on PTSD, a critical literature review suggested that dynamic approaches may be particularly suited to target the impact of trauma on crucial areas of interpersonal functioning, self-esteem, and affect tolerance [33]. The mechanisms of change in relational dynamic trauma therapy have been found effective in other therapy contexts. For instance, increases in insight or self-awareness are associated with improved interpersonal functioning and symptom severity [34]. The therapist’s facilitation of the patient’s affective experience and expression are positively associated with psychotherapy outcomes [35]. Increases in patients’ abilities to understand themselves and others in terms of mental states and subjective experiences are also associated with improved functioning [36]. Relational dynamic therapies are, by theory and in practice, not diagnostically bound or symptom-focused and thus may be a good fit in the rehabilitation model of recovery.

### Study Aims and Hypotheses

This project will develop a depth-oriented group psychotherapy for morally injured combat veterans. The research plan will use modern user-centered design methods that continuously gather user experiences during treatment development, with the goal of increased effectiveness and usability [25]. User feedback will be synthesized with formative feedback from a clinical expert panel. This objective will be accomplished by pursuing these specific aims.

#### Aim 1

Discover user needs and preferences as well as treatment-engagement barriers and facilitators from the perspectives of PMIE-impacted veterans and VA trauma clinicians (ie, mental health providers and chaplains).

#### Aim 2

Design a treatment manual and refine it using feedback from veterans, trauma clinicians, and an expert clinical advisory board.

#### Aim 3

Conduct 2 rapid prototyping open trials (ie, tangibly testing treatment approaches using a prototype manual) with PMIE-impacted veterans (N=12), and iteratively revise the manual based on veteran, provider, and clinical expert panel feedback, with the following hypothesis: “The treatment manual will meet usability, feasibility, learnability, and acceptability criteria.”

## Methods

### Study Design and Overview

This study design is a multiphase mixed methods treatment development project that involves (1) interviewing veterans and clinicians (Aim 1); (2) writing a treatment manual (Aim 2); and (3) open pilot testing the treatment manual in 2 successive psychotherapy groups (Aim 3). This project will use the user-centered DDBT framework to generate a treatment manual to treat the lasting and varied multidimensional functional impairments associated with high-magnitude PMIEs in a group psychotherapy context. We plan for approximately 50 Central Texas Veterans Healthcare System (CTVHCS) veterans to complete study activities: up to 38 veterans will complete Aim 1 interviews, and approximately 12 veterans will complete Aim 3 group psychotherapy pilot trials and associated assessments. See Table 2 for the timeline of study activities. See Table 3 for a data summary.

**Table 2.** Gantt table of study activities and timeline.

Gantt table	Year 1				Year 2				Year 3				Year 4				Year 5			
Quarter	1	2	3	4	1	2	3	4	1	2	3	4	1	2	3	4	1	2	3	4
Aim 1: Discover user needs as well as treatment barriers and facilitators from the perspectives of PMIE^a^-impacted transitioning veterans and VA^b^ providers.																				
Interview PMIE-exposed veterans (n=20)	✓	✓	✓	✓																
Interview VA trauma clinicians (n=20)	✓	✓	✓	✓																
Interview past participants of PI’s^c^ therapy group (n=18)	✓	✓	✓	✓																
Qualitative data analysis	✓	✓	✓	✓																
Aim 2: Design and build a treatment manual based on Aim 1 findings, clinician feedback, and depth-oriented relational dynamic research and practice.																				
Design and build a group therapy to treat PMIE-related functional and social impairments	✓	✓	✓	✓	✓	✓	✓	✓												
Reinterview VA trauma clinicians for feedback (n=20)						✓	✓													
Clinical expert review panel convenes	✓		✓		✓		✓													
Pilot trial startup procedures (eg, clinic coordination)								✓												
Aim 3: test the treatment manual via iterative open pilot trials with PMIE-impacted veterans.																				
First open pilot									✓	✓	✓	✓								
Interventionist training (2 interventionists)									✓											
Recruitment, screening, and consent (n=6)									✓											
Treatment and supervision (2 interventionists)										✓	✓									
Data collection, analysis, and interpretation										✓	✓	✓		✓						
Clinical expert panel reviews treatment manual												✓								
Treatment manual revision												✓	✓							
Second open pilot														✓	✓	✓	✓			
Recruitment, screening, and consent (n=6)														✓						
Treatment and supervision (2 interventionists)															✓	✓				
Data collection, analysis, and interpretation															✓	✓	✓		✓	
Revision of treatment manual																	✓	✓	✓	✓
Develop a fidelity and competency scale																	✓	✓		
Clinical expert panel reviews manual and new scale																		✓		

a PMIE: potentially morally injurious event.

b VA: Department of Veterans Affairs.

c PI: principal investigator

**Table 3.** Summary of data collection and analysis.

User-centered design phase, participants, and role		Method and timing of assessment	Variables and topics	Analysis plan	Study aim
Qualitative data					
Discover					
Veterans: potential future users		One-time, one hour semistructured interview	Needs Preferences (eg, group vs individual) Barriers and facilitators to participation	Rapid qualitative analysis	Aim 1
Clinicians and potential future users		—^a^	Needs Preferences	—	Aim 1
Veterans: former users		—	Experiences Preferences Areas for improvement	—	Aim 1
Design and build					
Clinical expert review panel		Biannual semistructured focus group, in Years 1‐3	Techniques Goals Format Active ingredients		Aim 2
Clinicians and potential future users		Think aloud review of treatment manual, via one-time one-hour semistructured interview	Usability issues Future context		Aim 2
Test					
Veterans and pilot trial participants		One-time, one-hour semistructured interview	Acceptability Usability		Aim 3
Clinicians and Interventionists		Feedback during supervision	Usability Learnability Acceptability		
Quantitative data					
Veterans and pilot trial participants		Planned versus actual recruitment Number of completed sessions Number of dropouts Self-report measures completed 1-month and 6-month posttreatment Clinical measure	Pilot trial primary outcomes: Usability Feasibility Acceptability Pilot trial exploratory clinical outcomes	Descriptive statistics: Mean SD 95% CIs Standardized mean change, for exploratory clinical outcomes	Aim 3

a Not applicable.

### Aim 1 Overview: Discover User Needs

The goal of Aim 1 is to discover user needs as well as potential barriers, facilitators, and structures of treatment from stakeholder perspectives. This process will involve user-centered interviews with (1) PMIE-impaired veterans in CTVHCS, (2) a national sample of VA trauma clinicians, and (3) previous participants in SO’s moral injury therapy group, a diverse set of VA mental health providers. The goal is to generate data on acceptability- and usability-related issues that will guide treatment development. These data will guide the design of the treatment manual in Aim 2.

### Aim 1 Interviews With PMIE-Impaired Veterans (n=20)

#### Participants

We will interview up to 20 US combat veterans enrolled in CTVHCS who report exposure to high-magnitude PMIEs and distress or functional impairment associated with the PMIE exposure (n=20).

#### Study Setting

Veterans will be recruited from CTVHCS. CTVHCS serves one of the largest veteran communities in the United States, as most veterans settle in and around the Fort Hood area following separation. For example, in 2014, approximately 125,000 veterans settled in Austin and the surrounding counties. Interviews will be conducted either in person or via an institutional review board (IRB)–approved teleconference.

#### Recruitment

We will use three strategies for recruiting participants.

1. Direct outreach to potentially eligible veterans: the names and contact information for potentially eligible veterans will be extracted from the VA Corporate Data Warehouse (CDW) via an approved Data Access Request Tracker (DART) request. CDW data requests will be limited to male and female, English-speaking veterans aged 18 years or older, enrolled in CTVHCS, and with a service record of combat deployments. Study staff will contact these veterans via postal mail with information about the study that includes a preaddressed, stamped postcard that they may return to opt out of receiving further contact about the study or express their interest in receiving a phone call from the investigator about the study. Staff will contact veterans that have not opted out and those who expressed interest in receiving more information via a VA office telephone or VA-issued, password-protected, and encrypted cellular phone 2 weeks after sending a study information letter to ask about their interest in the study.
2. Self-referral based on advertisements: a study brochure for participants will be placed in waiting rooms and with providers at CTVHCS hospitals and clinics.
3. Clinician referral: study staff will present the study aims and inclusion and exclusion criteria to CTVHCS providers in staff meetings and solicit referrals for the study. Study staff may send emails with study information to staff and may also contact staff via secure platforms (eg, encrypted VA email). Providers will be instructed to receive verbal permission from veterans to be contacted by study staff by phone and to document this in the veteran’s electronic medical record prior to sharing the veteran’s contact information with study staff.

#### Pre-Eligibility Screening

A trained study staffer will prescreen interested veterans for basic eligibility requirements: whether they served at least one combat deployment or received combat hazard pay; whether they are willing to answer questionnaires about distressing combat experiences, including morally injurious events, as part of eligibility screening; and whether they are willing to return study forms. Prescreening takes approximately 10 minutes.

#### Consent, Eligibility Screening, and Compensation

If veterans pass prescreening criteria, they will be scheduled for an informed consent and eligibility screening appointment. The potential participant will complete the informed consent prior to completing eligibility measures due to the sensitive content of these measures. To assess eligibility, participants will complete the event screener and brief Inventory of Psychosocial Functioning (b-IPF) [37] from the Moral Injury Outcome Scale (MIOS) [38], the Sheehan Disability Scale (SDS) [39], the Columbia-Suicide Severity Rating Scale (C-SSRS) [40], and the Montreal Cognitive Assessment (MoCA) [41]. See Table 4 for study measures. They will also be asked about their alcohol and substance use and dependence. The informed consent and eligibility screening will take approximately 45‐60 minutes. If participants provide consent and are eligible, then they will complete the full MIOS measure; if veterans are not eligible, we will not administer these items to reduce participant burden. At the end of the informed consent and eligibility screening, veterans will be scheduled for a one-hour interview with members of the study team. Veterans will be compensated US $30 after completing this one-hour interview. Veterans who decline to participate in the study but report an interest in mental health treatment will receive appropriate referrals.

**Table 4.** Table of veteran participant study measures.

Measurement information		Study aim 1: samples		Study aim 3			
Purpose and measure	Construct	Sample 1^a^	Sample 3^b^	Time points	Baseline	1month PG^c^	6 months PG
Eligibility (Aim 1, sample 1 and 3); descriptive (Aim 1, Sample 3)							
MIOS^d^ event screener	PMIE^e^ exposure	✓		✓		✓	✓
Eligibility, descriptive (Aim 1), exploratory outcome (Aim 3)							
SDS^f^	Functional impairment	✓		✓		✓	✓
b-IPF^g^, in MIOS	Psychosocial functioning	✓	✓	✓		✓	✓
Eligibility and safety							
C-SSRS^h^	Suicide risk	✓	✓	✓	✓	✓	
Eligibility							
MoCA^i^	Cognitive impairment	✓		✓			
Primary outcome (Aim 3)							
AIM^j^	Acceptability					✓	✓
IAM^k^	Implementability					✓	✓
FIM^l^	Feasibility					✓	✓
Descriptive (Aim 1); exploratory outcome (Aim 3)							
MIOS Outcome	Moral injury	✓	✓		✓	✓	✓
Exploratory outcome (Aim 3)							
PCL-5^m^	PTSD^n^				✓	✓	✓
BDI-II^o^	Depression				✓	✓	✓
BSS^p^	Suicidal ideation				✓	✓	✓
RSSS^q^	Spiritual distress				✓	✓	✓
MIES^r^	PMIEs				✓	✓	✓
MIQM^s^	Moral injury				✓	✓	✓
EMIS^t^	Moral injury				✓	✓	✓
GCS-R^u^	Group cohesion					✓	✓

a Sample 1: potentially morally injurious events–impacted veterans.

b Sample 3: nonmanualized psychotherapy past patients.

c PG: postgroup.

d MIOS: Moral Injury Outcome Scale.

e PMIE: potentially morally injurious event.

f SDS: Sheehan Disability Scale.

g b-IPF: brief Inventory of Psychosocial Functioning.

h C-SSRS: Columbia-Suicide Severity Rating Scale. Also administered at 3-month postgroup.

i MoCA: Montreal Cognitive Assessment.

j AIM: Acceptability of Implementation Measure.

k IAM: Implementation Appropriateness Measure.

l FIM: Feasibility of Intervention Measure.

m PCL-5: Posttraumatic Stress Disorder Checklist for *DSM-5* (*Diagnostic and Statistical Manual of Mental Disorders* [Fifth Edition]).

n PTSD: posttraumatic stress disorder.

o BDI-II: Beck Depression Inventory-II.

p BSS: Beck Scale for Suicide Ideation.

q RSSS: Religious and Spiritual Struggles Scale.

r MIES: Moral Injury Events Scale.

s MIQM: Moral Injury Questionnaire–Military version.

t EMIS: Expressions of Moral Injury Scale.

u GCS-R: Group Cohesion Scale–Revised.

#### Inclusion and Exclusion Criteria

Inclusion criteria are male and female, English-speaking veterans, aged 18 years or older, enrolled in CTVHCS, with a service history of combat deployments or combat hazard pay. Participants must (1) comprehend and sign the informed consent form; (2) report a history of PMIEs, identified by the event screener of the MIOS; and (3) report functional impairment secondary to their PMIE, as identified by ≥3 on the MIOS functional impairment items and ≥10 on the SDS. We believe that it is best to let exposure to life-span fear-based traumatic events freely vary among our participants. The concern is that excluding danger-based exposures in war veterans will unduly bias our study group. The MIOS event screen asks participants to endorse their worst and most currently distressing PMIE, which increases the likelihood that participants’ reports of moral injury will be based on an event that currently haunts and consumes them. Because we are looking for a range of PMIE-related impact, participants are not otherwise included or excluded for interviews based on psychiatric diagnoses. Exclusion criteria are substance abuse or dependence (other than caffeine or tobacco dependence); prospective participants who have maintained sobriety for at least 6 weeks prior to the time of enrollment may be eligible. Veterans will be excluded if they are deemed to be at high risk of suicide; veterans who are deemed at low or medium risk of suicide are considered eligible for the study. Suicide risk is evaluated using the C-SSRS using procedures outlined in the Risk Assessment Plan (see below). The C-SSRS is the VA-mandated suicide screening tool used in primary care and mental health clinics across the VA. If immediate clinical attention is warranted, veterans will be provided care consistent with procedures outlined in the Risk Assessment Plan. Veterans may also be excluded if they report evidence of cognitive impairment as identified by MoCA scores ≤17.

#### Risk Assessment Plan

We will follow CTVHCS Memorandum 116A-005 (latest version), “Suicide Prevention and Management of Suicidal Behavior.” Participants complete the C-SSRS [40] to assess suicide risk. The C-SSRS is routinely used in VA clinical care. Participants who screen negatively on the C-SSRS will be monitored throughout the course of the study. Participants who screen positive on the C-SSRS will have a warm hand-off to a licensed independent provider (LIP) or appropriate supervised trainee for a same-day comprehensive suicide risk evaluation. A comprehensive suicide risk evaluation results in an assessment of acute and chronic suicide risk as either low, medium, or high and suggests appropriate treatment for each risk level. Treatment planning and triage to the appropriate level of care will be conducted by the licensed independent provider and/or supervised trainee.

#### Sample Size Justification

The recommended sample size for usability studies in the discovery phase ranges from 3‐20 participants [42]. When the study is complex and the context is critical to consider, relatively larger sample sizes are recommended. Because of the novelty of the research and the complexity of PMIE-related impacts and the proposed treatment context, we plan to recruit up to 20 veterans. Usability study sample size recommendations (ie, the point at which additional interviews do not contribute new data or theoretical insights) are consistent with other qualitative research methods (n=16‐24) [43].

#### Data Collection

Eligible veterans will participate in a one-time, 1-hour-long interview about their needs and preferences for moral injury group psychotherapy, such as responses to a group versus individual format, treatment length and format, and potential barriers or facilitators to their potential participation; additionally, we will ask about past attempts to cope with the impact of PMIEs.

#### Data Analysis Plan

We will use the rapid qualitative analysis (RQA) for qualitative research to conduct interviews and analyze data [44]. RQA is an established, pragmatic, team-based approach for qualitative data that quickly generates preliminary findings to inform real-time modifications to an implementation strategy. RQA involves (1) creating a brief, prioritized semistructured interview guide; (2) reducing each interview question to a simple domain name; (3) creating a summary table to facilitate data reduction in which the key points from each interview are summarized under the relevant domain name; (4) transferring summary points to data matrix; and (5) conducting matrix analysis to quickly assess the content of interview domains and any gaps in information as well as to develop cross-interview summaries of themes and types of responses.

### Aim 1 User Need Interviews With VA Trauma Clinicians (N=~20)

#### Participants

Up to 20 VA trauma clinicians will be recruited for a one-time, 1-hour semistructured interview about their perceived needs, barriers, structures, and facilitators of conducting a PMIE-focused group treatment. We plan to recruit psychologists, psychiatrists, social workers, peer support specialists, and chaplains who provide trauma-focused mental health treatment.

#### Study Setting

Interviews with VA trauma clinicians will occur via teleconference because a national sample of providers will be recruited.

#### Recruitment and Consent

The study team (eg, PI, consultants, and clinical expert panel) and professional networks will help refer and recruit trauma clinicians from across the VA. The recruitment process will occur in three stages: (1) an IRB-approved broad study recruitment email will be disseminated by the study team to colleagues and professional networks. This email will have a deadline by which date VA clinicians can indicate their tentative interest. (2) the PI will submit an IRB amendment adding each clinician’s VA facility as a study site. (3) In tandem with (2), PI will submit the list of VA facilities to the American Federation of Government Employees (AFGE) National Union. The AFGE National Union office will coordinate local facility approval. IRB and AFGE approval will be obtained prior to enrolling and consenting clinicians. Once IRB and AFGE approvals are obtained, the PI will formally contact the clinicians who indicated interest in participation. Clinicians who participate in Aim 1 interviews will be encouraged to reach out to their colleagues and will be sent the recruitment email with an updated deadline. Clinicians will be emailed a digital consent form via encrypted email and will be asked to digitally sign and return the consent form prior to the teleconference interviews. VA employees will not be compensated for their participation.

#### Inclusion/Exclusion Criteria

Potential participants will include male and female VA providers who treat veterans for trauma-related mental health services. Clinicians will be recruited from across the VA and not be limited to CTVHCS providers. Participants must be able to provide written or digital consent to participate. Providers must also agree to be recontacted to provide feedback on a draft of the treatment manual in order to participate.

#### Sample Size Justification

We aim to recruit 20 providers because this number is recommended in the discovery stage phase of usability studies [42].

#### Data Collection, Analysis, and Interpretation Plan

Interviews will last approximately one hour. Interviews will be conducted, and data will be analyzed using the RQA for qualitative data. See above for description of RQA procedures.

### Conduct Aim 1 Interviews With Past Members of the PI’s Nonmanualized Moral Injury Group Psychotherapy

### Participants

Veterans who had participated in the PI’s nonmanualized moral injury group psychotherapy (n=up to 18) will be invited to participate in one-time, 1-hour-long interviews. The goal is to learn what in these past patients’ group psychotherapy experience seemed to resonate with or help them, or not. We also hope to learn what past patients would want future veteran patients to know or experience in a moral injury group psychotherapy.

#### Study Setting

Interviews with past patients will occur either in-person at a CTVHCS mental health clinic or via teleconference.

#### Recruitment and Eligibility Prescreening

Study staff will contact past patients via postal mail with information about the study that includes a preaddressed stamped postcard that they may return to opt out of receiving further contact about the study or express their interest in receiving a phone call from the investigator about the study. After 2 weeks of sending the study information letter, staff will contact those veterans that have not opted out and those who expressed interest via telephone.

A trained study staff member will prescreen veterans for basic eligibility requirements: whether they are interested in participating in a 1-hour interview about their previous therapy experiences and whether they are willing to send back signed study forms. Prescreening will take approximately 10 minutes.

#### Consent, Eligibility Screening, and Compensation

If veterans pass prescreen criteria, they will be scheduled for an informed consent and eligibility screening appointment. Potential participants will provide informed consent prior to completing eligibility screening measures. To assess eligibility, veterans will complete the C-SSRS [40]. The informed consent and eligibility screening will take approximately 30‐45 minutes. If veterans provide consent and are deemed eligible, they will be scheduled for a one-time, 1-hour interview. Veterans will be compensated US $30 after completing this interview. Veterans who consent to participate in the study will also be administered the MIOS [38]. See Table 4 for study measures. Veterans who decline to participate but report an interest in mental health treatment will be referred for follow-up care.

#### Inclusion/Exclusion Criteria

All past group members, including veterans who did not complete the therapy, will be contacted and offered an opportunity to participate. Veterans who are at low or medium risk of suicide are considered eligible, and veterans who are deemed at high risk of suicide are not eligible and will be triaged to the appropriate level of care. Suicide risk will be evaluated with the risk management plan (see below).

#### Sample Size Justification

Our sample size is constrained by the number of previous group members who agree to participate in interviews. Usability study sample size guidelines vary from 3 to 20 participants [42]; thus, we are likely to obtain meaningful results if only a minority of previous members participate.

#### Data Collection, Analysis, and Interpretation Plan

Interviews will be conducted and data analyzed using the rapid assessment process for qualitative data [44].

### Aim 2 Overview: Design and “Build” a Treatment Manual

#### Overview

The goal of Aim 2 is to design and build (ie, write) a treatment manual to treat the lasting and varied multidimensional impact of PMIEs. The treatment manual will be written based on Aim 1 findings, clinical evidence and theory, and feedback from the clinical expert panel. User-centered design suggests that, in the design phase, important tasks involve synthesizing user needs, developing a low-fidelity prototype, and gathering usability data from potential users (eg, providers who may use the manual).

The PI will lead treatment manual writing in close consultation with coinvestigators, consultants, and the clinical expert panel. The manual will be iteratively revised based on data gathered from Aim 1 interviews, the clinical expert panel, and once the pilot trial commences, feedback from pilot trial participants.

#### Clinical Expert Panel Feedback on the Treatment Manual

A panel of expert clinicians will convene to provide guidance on the development of the treatment manual, including reviewing and providing constructive criticism on iterative drafts of the manual. The clinical expert panel will provide a perspective on the usability and learnability of the manual for nonrelational dynamic future users, in particular. The clinical expert panel may also suggest new approaches, which will be discussed with the study team. Importantly, the clinical expert panel will not receive any sensitive or identifiable data.

#### Qualitative Data Analysis Plan

Clinical expert panel meetings will be conducted and data analyzed using the RQA for qualitative data [44].

#### Reinterview VA Trauma Clinicians to Gather Feedback on the Treatment Manual (n=~20)

We will recontact the clinicians who participated in the Aim 1 interview to obtain their feedback on drafts of the treatment manual. Interviews with VA trauma clinicians will occur via teleconference. Interviews will be conducted, and data will be analyzed using RQA for qualitative data [44]. We will use the think-aloud strategy to guide semistructured interviews. The think-aloud method involves potential users reviewing the treatment manual while simultaneously and continuously speaking about their reactions, which the researchers record. The think-aloud method is recommended by user-centered design experts for the design phase.

### Aim 3 Overview: Test the Treatment Manual via 2 Open Pilot Trials

#### Overview

The goal of Aim 3 is to test the treatment manual via 2 iterative open pilot trials with PMIE-impacted veterans. User-centered design principles suggest that an intervention should be rapidly prototyped, piloted, and revised based on user feedback [25,45]. To achieve this aim, we will run two pilot trials of the PMIE-focused treatment manual in the CTVHCS mental health clinic. Data on usability, learnability, feasibility, and acceptability will be gathered from veterans and interventionists, and initial data on clinically meaningful outcomes will be gathered from veterans. The treatment manual will be revised at the end of the first pilot trial and will be finalized at the end of the second pilot trial.

#### Participants

We aim to enroll approximately 12 PMIE-impacted US combat veterans total into 2 open pilot trials; approximately 6 veterans will be enrolled in each pilot trial.

#### Study Site

The pilot trials will be conducted in the CTVHCS mental health clinics. The mental health clinic is staffed by multidisciplinary care teams that provide comprehensive mental and behavioral health treatments, including psychosocial rehabilitation, substance use treatment, trauma-focused treatment, and chronic pain and physical disability-related treatments, as well as general mental health treatment.

#### Recruitment, Pre-Eligibility Screening, Consent, and Eligibility Screening

We will use the same methods for recruitment, pre-eligibility screening, consent, eligibility screening, and compensation, as used in Aim 1 interviews with PMIE-impacted veterans. See description of Aim 1 methods for details. See Table 4 for eligibility screening measures.

#### Inclusion/Exclusion Criteria

We will use the same inclusion and exclusion criteria for the pilot trials as used in Aim 1 interviews with PMIE-impacted veterans. This will increase the likelihood that user-centered recommendations will fit the intended treatment sample.

#### Sample Size Justification

We aim for 6 to 8 participants per group based on recommendations for group therapy sizes; we will recruit up to ten participants per group to account for expected dropout [22].

#### Treatment Delivery and Supervision

The PMIE treatment manual designed in Aim 2 will be delivered to 2 successive open pilot groups. Based on the PI’s experience providing nonmanualized PMIE-focused group psychotherapy and clinical literature that suggests more sessions are needed or complex psychological problems [46], we expect the group to be approximately 25 weekly sessions. Each session will be 90 minutes each. The group will be led by 2 trained mental health providers. Procedures for identifying and handling treatment dropouts (eg, how many missed sessions until a participant is considered “dropped out”) will be developed as part of this pilot trial. Participants that cease attending treatment sessions will be recontacted to complete follow-up assessments. Interventionists will receive ongoing supervision via regularly scheduled teleconferences.

#### Risk Assessment Plan

Participants will receive 3 follow-up check-in phone calls following the end of the treatments: the first at one-month posttreatment, the second at 3-month posttreatment, and the third at 6-month posttreatment. During these follow-up phone calls, participants’ suicide risk will be assessed using the C-SSRS. We will follow the CTVHCS Memorandum 116A-005 (the latest version), “Suicide Prevention and Management of Suicidal Behavior,” to guide risk assessment and treatment planning. All participants, regardless of response on the C-SSRS, will be offered referrals to mental health services as requested or needed.

#### Study Measures and Participant Characterization

See Table 4 for study measures, assessment time points, and participant pay. Most study measures are self-report and will be completed via VA REDcap (Research Electronic Data Capture; Vanderbilt University) survey. Participants will be characterized based on demographic and medical history data, including data collected via the VA electronic health record: age; ethnicity; psychiatric treatment history, including recent psychiatric admission; medications and medical history; years of education; marital status; housing; disability and employment status; history of childhood trauma; and past and current mental health diagnoses in the electronic health record problem list.

Participants will complete study measures at 3 time points: a baseline assessment prior to group psychotherapy, following the end of the group therapy (approximately 6 months postbaseline), and 6 months after the end of the group psychotherapy (approximately 12 months postbaseline). Participants will also be called approximately 3 months after the end of group psychotherapy for a clinical check-in; at this time point, only the C-SSRS will be administered. Participants will be contacted via IRB-approved methods up to 3 times to complete each assessment.

#### Primary Outcome Measures: Feasibility and Acceptability

Acceptability and feasibility will be assessed in several ways: (1) actual recruitment versus planned recruitment; (2) participants’ number of sessions completed and number of dropouts; (3) qualitative interview data from interventionists, collected by the PI during weekly supervision sessions, regarding reactions to the treatment manual and satisfaction with the treatment manual; and (4) qualitative interview data from veterans on participants’ enthusiasm for the therapy, comprehension of therapy, and application of therapy interventions, collected by the PI or approved study team member during follow-up exploratory outcome assessment phone calls. Veterans and providers will also complete three 4-item measures that were developed by the user-centered design methodologists: Acceptability of Implementation Measure (AIM), Implementation Appropriateness Measure (IAM), and the Feasibility of Intervention Measure (FIM) [47]. The AIM, IAM, and FIM scores demonstrated adequate reliability and validity in initial psychometric testing in samples of mental health professionals. Usability and learnability will be assessed via the Intervention Usability Scale (IUS) and qualitatively gathered feedback from SO and GB. The 10-item IUS was adapted from the widely used System Usability Scale. It is scored on a Likert-style scale ranging from 0 (strongly disagree) to 4 (strongly agree) and provides a percentile ranking of usability.

#### Exploratory Clinical Outcomes

The primary exploratory outcome is functional impairment, assessed using the SDS [39]. We will also collect exploratory data on secondary outcomes. PTSD will be measured with the widely used 20-item Posttraumatic Stress Disorder Checklist for *DSM-5* (*Diagnostic and Statistical Manual of Mental Disorders* [Fifth Edition]; PCL-5), which assesses severity of each *DSM-5* PTSD symptom using a Likert-style scale ranging from 0 (not at all) to 4 (extremely) [48]. A total score of 31‐33 is suggestive of a PTSD diagnosis. The PCL-5 demonstrated Cronbach α=0.95 in a sample of veterans in the VA [49]. Depression will be measured with the 21-item Beck Depression Inventory-II (BDI-II), which uses a Likert-style scale ranging from 0 to 3 [50]. The BDI-II is widely used, with Cronbach α=0.92 in samples of adults. Suicidal ideation will be measured using the Beck Scale for Suicide Ideation (BSS), which is extensively used in treatment studies of suicidal individuals [51]. The BSS is rated on a Likert-style scale ranging from 0 to 3, and scores have Cronbach α>0.90 in both inpatient and outpatient settings. Spiritual distress will be measured with the 26-item Religious and Spiritual Struggles Scale (RSSS), which assesses 6 domains, including moral and doubt struggles, using a Likert-style scale ranging from 0 to 5 [52]. We will also collect data on group cohesion to examine group-specific contributions to outcomes using the 25-item Group Cohesion Scale-Revised [53], which measures group functioning on a Likert-style scale ranging from 0 (low) to 4.

We will also collect exploratory data on moral injury-specific outcomes using the MIOS [38], the 9-item Moral Injury Events Scale (MIES) [54], the 20-item Moral Injury Questionnaire–Military version (MIQM) [55], and the 17-item Expressions of Moral Injury Scale (EMIS) [56]. Data on the MIOS, MIES, MIQM, and EMIS will also allow results from this study to cross-talk with external efforts to map and treat the impact of moral injury, such as the VA-funded ACT for Moral Injury trial (RX002854-01A1).

### Data Analysis Plan

#### Quantitative Data

All quantitative data will be entered into an IRB-approved electronic database (eg, SPSS [IBM Corp], REDCap, Excel [Microsoft Corp]) constructed by the PI or study team member. Quantitative data analysis will occur on the approved research drive or the VA Informatics and Computing Infrastructure (VINCI) [57] cloud computing software server. Tenets of our data system include double data entry (by PI and study coordinator), range checks, and/or exclusion of identifiers that can be traced to individuals. Preliminary analyses will include computing descriptive statistics, inspecting for normality of data, and, if necessary, transforming data.

#### Missing Data

Missing data will be considered a valuable source of information about the feasibility of the intervention and the study procedures, consistent with the appropriate role of clinical research pilot studies [58]. Nonattendance at group psychotherapy sessions, including dropout, will be used to calculate feasibility outcomes. Missing data on primary and exploratory clinical outcome measures will be handled using pairwise deletion.

#### Feasibility, Acceptability, and Usability

We will assess the feasibility of (1) recruitment, (2) attendance and retention, and (3) ease of implementation. To examine potential effects of individual differences on the feasibility of recruitment rates and attendance and retention, we will explore whether patterns of any demographic features (eg, age, living situation, and employment status) are associated with successful recruitment and attendance rates. We will include these variables as covariates in our analyses when appropriate. For recruitment and attendance, we will estimate the proportion of participants who have adequate recruitment and attendance rates with a 95% CI around the proportion. Recruitment will be deemed successful if >75% of veterans referred to the PMIE-focused treatment agree to participate. Attendance and retention will be deemed successful if 75% of veterans attend >75% of sessions. The AIM, IAM, and FIM will be descriptively analyzed, and the mean and SD for the initial session and for the final session will be reported; mean scores ≥4 will indicate acceptability (via the AIM), appropriateness (via the IAM), or feasibility (via the FIM). The IUS generates a percentile ranking, and scores >68 are considered above average.

#### Exploratory Clinical Outcomes

Formal hypothesis testing is inappropriate in pilot studies because small sample sizes are likely to lead to inaccurate estimates, and pilot studies test an evolving intervention and clinical training rather than a final product [58]. We will calculate and report mean scores with 95% CIs of clinically meaningful outcomes, consistent with CONSORT (Consolidated Standards of Reporting Trials) reporting guidelines [59].

### Qualitative Data on Feasibility, Acceptability, and Usability

Qualitative data will be analyzed using the RQA methods [44].

### Fidelity and Competency Measure Development

We will develop a fidelity and competency measure in preparation for a full-scale randomized controlled clinical trial. Measure development procedures will be based on the “Field Guide to Developing a Comprehensive Fidelity Measurement System” [60]. The fidelity and competency measure will assess the essential components of the content and process of the moral injury group psychotherapy and its delivery. This measure will be presented to the clinical expert panel for review and will be subsequently revised based on their feedback.

### Ethical Considerations

CTVHCS Institutional Review Board approval was obtained prior to study initiation (study project #2021‐007). We will obtain written informed consent from all participants prior to engaging in research study activities. The privacy and confidentiality of research subjects’ data and/or identity are maintained in accordance with relevant local and national policies and guidance. Veteran participants were paid approximately US $30 per hour for participation in research assessments; VA employees were not compensated for their participation.

## Results

This study was funded with a start date of November 2021. Participant recruitment for Aim 3 pilot trials began in October 2023 and ended in January 2025. We anticipate study data collection to be complete in January 2026 and primary data analysis to be completed by April 2026.

## Discussion

The goal of this project is to develop and pilot test a treatment manual for a novel depth-oriented group psychotherapy for morally injured combat veterans. We anticipate that the manual will meet predetermined usability, feasibility, and acceptability criteria; we will also explore the potential for impact on relevant clinical outcomes, including functional impairment. We will use user-centered principles of treatment design to meet our usability and feasibility goals. If the treatment does not demonstrate preliminary usability or feasibility by the second pilot trial, we will evaluate user feedback to better understand treatment barriers. We plan to revise the treatment manual to enhance usability, learnability, and fit because we intentionally gathered participant feedback at each stage of treatment development, including after the second pilot trial.

This study is marked by many strengths as well as some limitations. Our pilot trial is well-poised to examine multiple aspects of intervention and trial feasibility, including screening and recruitment processes, treatment retention rates, and the assessment process. This pilot trial process will allow the study team to develop good clinical practices and training procedures to prepare for a future efficacy trial. We decided to run open pilot trials rather than randomized controlled pilot trials because of resource considerations; the lack of a comparator group, however, may limit some of the conclusions we may draw about study procedures.

This study will guide future efforts to refine treatments for moral injury as well as potentially seed a future efficacy trial of the treatment manual. Should the current intervention meet the primary outcomes, our next step will be to design and execute a parallel group randomized controlled clinical trial of the moral injury group psychotherapy intervention. Additionally, we plan to disseminate learnings from Aim 1 interviews with regard to combat veteran and VA clinician needs, preferences, and barriers to moral injury psychotherapy and, in particular, group psychotherapy. These data may inform other treatment developers’ efforts to intervene on moral injury as well. Veterans have unique needs and experiences that require culturally responsive and sensitive conceptualizations and treatments. Patient-centered care is improved by providing multiple effective treatment options.

## Supplementary material

10.2196/87756 Checklist 1 SPIRIT checklist.

10.2196/87756 Peer Review Report 1 Peer review report from RRD8 Career Development Program - Panel I, Office of Research and Development, Rehabilitation Research and Development (Department of Veterans Affairs, USA)

## Abbreviations

ACT-MI: acceptance and commitment therapy for moral injury
AD: adaptive disclosure
AFGE: American Federation of Government Employee
AIM: Acceptability of Implementation Measure
b-IPF: brief Inventory of Psychosocial Functioning
BDI-II: Beck Depression Inventory-II
BSS: Beck Scale for Suicide Ideation
C-SSRS: Columbia-Suicide Severity Rating Scale
CDW: Corporate Data Warehouse
CONSORT: Consolidated Standards of Reporting Trials
CPT: cognitive processing therapy
CTVHCS: Central Texas Veterans Healthcare System
DART: Data Access Request Tracker
DDBT: Discover, Design and Build, and Test
DoD: Department of Defense
*DSM-5*: *Diagnostic and Statistical Manual of Mental Disorders* (Fifth Edition)
EMIS: Expressions of Moral Injury Scale
FIM: Feasibility of Intervention Measure
IAM: Implementation Appropriateness Measure
IOK: impact of killing
IRB: institutional review board
IUS: Intervention Usability Scale
LIP: licensed independent provider
MIES: Moral Injury Events Scale
MIOS: Moral Injury Outcome Scale
MIQM: 20-item Moral Injury Questionnaire–Military version
MoCA: Montreal Cognitive Assessment
OEF: Operation Enduring Freedom
OIF: Operation Iraqi Freedom
PCL-5: Posttraumatic Stress Disorder Checklist for *DSM-5*
PE: prolonged exposure
PI: principal investigator
PMIE: potentially morally injurious event
PTSD: posttraumatic stress disorder
REDCap: Research Electronic Data Capture
RQA: rapid qualitative analysis
RSSS: Religious and Spiritual Struggles Scale
SDS: Sheehan Disability Scale
VA: Department of Veterans Affairs
VINCI: VA Informatics and Computing Infrastructure
