# Supplementary material for: Development of a Group Psychotherapy for Combat Veterans With Moral Injury: Protocol for a User-Centered Study
Source: JMIR Res Protoc. 2026 May 27;15:e87756. doi: 10.2196/87756 (PMC13215664; doi:10.2196/87756)
Supplement: Peer Review Report 1 [file resprot-v15-e87756-s002.pdf]

SUMMARY STATEMENT

**PROGRAM CONTACT:**	**(Privileged Communication)**	***Release Date:***	**03/22/2021**
		Revised Date:	

Application Number: **1 IK2 RX003495-01A2**

Principal Investigator ***Formerly:*** **1IK2RX003495-01A1**

FRANKFURT, SHEILA

Applicant Organization: OLIN TEAGUE VETERANS CENTER

Review Group: **RRD8**
Career Development Program - Panel I
RRD8

Meeting Date: **02/22/2021** ***RFA/PA:*** **RX20-006**
Council: **MAY 2021**
Requested Start: **09/01/2021**

Project Title: **Consulting after Combat: Interviewing Service Members and Veterans to Develop a Therapy to Restore Functioning and Reintegration after Moral Injury Events**
SRG Action: **Impact Score:125**

Human Subjects: **30-Human subjects involved - Certified, no SRG concerns**
Animal Subjects: **10-No live vertebrate animals involved for competing appl.**
Gender: **1A-Both genders, scientifically acceptable**
Minority: **1A-Minorities and non-minorities, scientifically acceptable**
Age: **1A-Children, Adults, Older Adults, scientifically acceptable**
Clinical Research - not NIH-defined Phase III Trial

Project Year	Direct Costs Requested
1	209,306
2	214,343
3	219,471
4	226,178
5	229,586
TOTAL	1,098,884

ADMINISTRATIVE BUDGET NOTE: The budget shown is the requested budget and has not been adjusted to reflect any recommendations made by reviewers. If an award is planned, the costs will be calculated by VA Office of Research and Development (ORD) staff based on the recommendations outlined in the BUDGET COMMENT section and any relevant ORD service-specific limitations.

SUMMARY OF DISCUSSION:
A Subcommittee of the Rehabilitation Research and Development Scientific Merit Review Board met in Plenary Session and reviewed the above proposal considering all internal and external reviews. This document summarizes the major points of the discussion concerning the proposed project. In any further development of this project, the investigator should consider carefully all the issues reflected in this Summary of Discussion as well as the more detailed comments in the individual critiques.

GENERAL COMMENTS:
Reviewers agreed that this CDA2 applicant has great potential to continue establishing herself as an independent researcher within the VA and that the training plan would effectively support this. Additionally, all reviewers commented on the significant public health relevance of moral injury and potential of a new moral injury therapy to improve VA care. Reviewers believed that the applicant had been very responsive to the last round of reviews and that this has resulted in an excellent scientific approach to treatment development. Reviewers noted some limitations related to the construct of moral injury and its differentiation from PTSD, as well as the somewhat limited empirical support for using relational dynamic therapy for trauma. However, it was agreed that these are limitations in the field of moral injury rather than specific or actionable weaknesses of the current proposal. No major weaknesses were noted.

SUGGESTIONS:
One reviewer raised the question of gender differences and how these would be addressed. Although the number of women in the study would likely be very small, it could be beneficial to assess for the potential impact of gender during qualitative interviews. It would also be useful to know more about potential differences in the groups when they are not limited to men. Reviewers noted that the sample would likely be too small for meaningful analyses of gender differences, but preliminary data could inform hypothesis generation for gender-related aims during a larger hybrid efficacy trial.

COMMENTS ON THE BUDGET:
The budget was believed to be appropriate.

ADMINISTRATIVE NOTE:
Recruitment table indicates that 50 Veterans will be recruited over the course of the study. No allowances are made for attrition. Please revise the recruitment table accordingly.

DESCRIPTION (provided by applicant):
Approximately 25% of combat Veterans with Posttraumatic Stress Disorder (PTSD) seek treatment for traumas that involve potentially morally injurious events (PMIE) rather than danger-based traumas. PMIEs are more strongly associated with functional and psychiatric impairment than life-threat-based combat. Veterans report that PMIEs disrupt their sense of identity and meaning, ability to connect with and trust others, and engender disturbing guilt, shame, rage, and disgust. The sequelae of exposure to PMIEs, otherwise known as moral injury, may explain variance in post-deployment recovery and is a potential unaddressed treatment target. Existing first-line treatments may be limited because they were derived from civilian contexts, poorly fit the war zone context, and do not allow Veterans to discuss the details of the PMIEs with other Veterans. This project will develop a relational dynamic-based group therapy treatment manual that will target functioning and quality of life among Veterans who are impacted by high magnitude PMIEs. The goal of this relational dynamic trauma therapy is to help Veterans identify connections between their current symptoms and their experiences in combat/PMIEs, their current life stressors and relationships, and the historical factors that carry person-specific meaning to their trauma/PMIE. These explorations take place in the presence of attuned and sympathetic others who can resonate to the experience and the affect being expressed. Symptom

reduction occurs through increasing the Veteran's capacity to consciously reflect on their experiences and develop an integrated self-awareness of the various factors that affect their mental states. The result is greater self-reflection, less avoidance, and greater adaptive incorporation of life experiences and their aftermath and meanings into one's inner world. This CDA2 will employ innovative user-centered design methods that continuously gather user experiences during treatment development, with the goal of increased effectiveness and usability. User feedback will be synthesized with formative feedback from a clinical expert panel. This objective will be accomplished by pursuing these specific aims: Aim 1: Discover user needs and preferences as well as treatment-engagement barriers and facilitators from the perspectives of PMIE-impacted Veterans and [VA trauma clinicians]. Aim 2: Design a treatment manual and refine it using feedback from Veterans, [trauma clinicians], and an expert clinical advisory board. Aim 3: Conduct two rapid prototyping open trials (i.e., tangibly testing treatment approaches using a prototype manual) with PMIE-impacted Veterans (N = ~12), and iteratively revise the manual based on Veteran, provider, and clinical expert panel feedback, with the following hypothesis: The treatment manual will meet usability, feasibility, learnability, and acceptability criteria.

PUBLIC HEALTH RELEVANCE:
Despite the VA's best efforts to treat the psychosocial impact of war, many combat Veterans report lingering difficulty reintegrating into meaningful post-deployment lives. War is among the most extreme forms of human experience but, for many, wartime trauma was treated using models transported from civilian single-incident trauma contexts. Veterans have unique needs and experiences that require culturally responsive and sensitive conceptualizations and treatments. Patient-centered care is improved by providing multiple effective treatment options and this project, if successful, could have a significant impact on VA care. This CDA2 project has the potential to offer innovative treatment for traumatized combat Veterans who otherwise may not find full relief from PTSD. Clinical research practice will be advanced by employing state-of-the-art user-centered design methods combined with expert clinical feedback to develop an effective and usable group treatment manual that will meet VA needs.

CRITIQUE 1

Applicant: Shelia Frankfurt. The applicant successfully completed the VA advanced fellowship for mental illness research and treatment and a CDA1. She shows a strong commitment to the VA. With 21 peer reviewed publications (10 first authored) and two moral injury book chapters, the applicant displays strong promise to develop into an independent clinical researcher. This is also reflected in her letters of support. The applicant has a track record of publishing on the topic of moral injury, including a first authored paper on moral injury, a commentary, and the above noted book chapters.

Mentor(s): Erin Finley (primary), Ghislaine Boulanger (Co), Marianne Goodman (Co), Brett Litz (Co)
Consultants: Joseph Geraci, Steven Nelson, Harold Kudler, Alejandro Interian, Thomas McGoldrick.

This is a strong mentoring team. The applicant has a track record of collaborating with the mentoring team and the team is well-suited to meet the applicant's training needs.

Training Program: The proposed training program is comprehensive and would help the applicant develop as a VA rehabilitation researcher. The training goals are aligned with furthering the applicant's expertise in moral injury and user centered design. The proposed training would also help her execute her plan to propose a hybrid design trial evaluating her intervention following completion of the CDA2.

Scientific Merit: The proposed study does not appear to be directly related to Dr. Finley's primary research projects. The work would definitely enable the applicant to develop her own research expertise and is adequately distinct from the mentors.

Significance: There is increasing empirical support that moral injury is a significant problem among Veterans. The impact of potentially morally injurious events has largely been overlooked in mental healthcare and moral injury treatments hold great promise for significantly impacting the wellbeing of Veterans. There are some existing interventions for moral injury, but most focus on single PMIEs or are individual interventions. ACT-MI is another group intervention being developed for moral injury, but this does not diminish the significance of the proposal because this reviewer believes that having another group moral injury intervention using a different therapeutic orientation would be beneficial for providing Veterans with treatment options and promoting patient centered care. The proposed intervention is unique in that it uses relational dynamic therapy and allows patients to discuss the details of their PMIEs. The proposed intervention could have a significant impact on VA care.

Approach: The applicant was very responsive to feedback and has strengthened the approach of the proposed research. The revised approach combines state-of-the-art methods from implementation science; patient, clinician, and clinical expert input; and iterative rounds of manual revisions based on user feedback with the goal of creating a well-developed effective intervention. This approach is a tremendous strength for creating an intervention that will be acceptable and ready for dissemination.

The qualitative sample of potential users is now more representative of potential patients and clinicians (focused on trauma clinicians).

Aspects of the timeline were improved.

The applicant responded to concerns about the operationalization of PMIE exposed Veterans and is now focusing on Veterans with deficits in functioning due to a MIE. She is using an approach with self-report measures that is likely to be reliable. This should result in a well-defined sample most likely to benefit from the intervention.

The change from attempting to develop a new measure to collecting data on the MIOS has increased feasibility.

The addition of developing a fidelity and competency scale has also strengthened the approach and will help prepare for a future hybrid trial.

The theory that underlies the proposed relational dynamic approach to treating moral injury is better articulated, but still appears to have fairly limited empirical support, which is a limitation of the proposed approach. This limitation is tempered somewhat by the fact that the absence of other relational dynamic therapies within the VA or for trauma treatment increase the innovation/significance of the proposal.

Resources: The facilities and resources available to carry out the proposed study are excellent. There is adequate demonstration of institutional support and the applicant has previous experience working in the proposed settings. The scientific environment is very strong and will contribute to the probability of success.

Protection of Human Subjects: Protections from research risk are adequate and the potential benefit of the proposed project is significant.

Inclusion of Women, Minorities, and Children: Proposed plans are adequate**.**

Critique of Vertebrate Animals Section:

	Yes	No
Research with vertebrate animals?		X

Biohazards and Radioisotopes: No comment.

Additional Review Criteria (unscored): No comment.

Budget (unscored)**:** Appropriate.

Data Management and Access Plan (for data sharing, unscored)**:** Appropriate.

Overall Strengths:
- Very responsive to reviews.
- Integrating user feedback and formative evaluation by experts.
- This CDA2 application is from a strong applicant who has shown dedication to a VA career and collaborated successfully with the proposed mentorship team.
- The mentorship team includes experts in treatment development and moral injury who have had successful clinical research careers in the VA.
- The training plan would prepare the applicant for success as an independent researcher and help her develop expertise in implementation science methodology and moral injury.
- The proposed project focuses on developing a treatment for moral injury, a significant issue among Veterans. Moral injury treatments have the potential to significantly improve VHA care.
- The proposed approach integrating stake holder input could enhance the usability and acceptability of the intervention and make the manual well-suited for dissemination.

Overall Weaknesses: Evidence supporting use of relational dynamic therapy for trauma is limited.

CRITIQUE 2

Applicant: Dr. Sheila Frankfurt CDA2, lots of experience.

Mentor(s): Drs. Finley, Nelson, Boulanger, Litz.

Training Program: Olin E. Teague Veterans Center.

Scientific Merit:

Significance: Potentially high-impact study for a yet to be recognized syndrome, Moral Injury.

Approach: User-centered design approach: will ask participants to speak of their worst, as well as most currently distressing PMIE, in the hope to increase reports on moral injury. Variation of the MI has been used in injury-focused treatment trials done by the applicant's supervisor.

Psychometrics that has been improved since last application: Moral Injury Outcome Scale (MIOS) and the Sheehan Disability Scale (SDS), which improved recently.

Longitudinal: patients who participated in previous studies will be included to inform development of the initial therapy manual. (this is a new adding since last submission).

Qualitative: rapid assessment process (RAP).

Resources: Great mentors and mentorship training included into the application. COVID-19 has been taken into consideration in the revised application.

Protection of Human Subjects: No issue.

Inclusion of Women, Minorities, and Children: No issue.

Critique of Vertebrate Animals Section: Not applicable.

	Yes	No
Research with vertebrate animals?		X

Biohazards and Radioisotopes: Not applicable.

Additional Review Criteria (unscored)

Budget (unscored)**:** Consistent with VA regulation.

Data Management and Access Plan (for data sharing, unscored)**:** No issue.

Overall Strengths: This application addresses a significant area of work using a novel approach that will contribute to the novelty of the still emerging research on MI. The mentoring team is very strong and well involved at every step of the program. The training plan addressed previous feedback and will lead the applicant very well to the next professional step of her already advanced career.

Sample (20) is acceptable but number of sessions (25) is quite impressive and should help refining the manual. Would clarify the role of former participants are they part of the 20.

Overall Weaknesses: Better define the relationship between PMIE and PTSD: how are they identified and distinguished for recruitment as participants? How will “co-morbidity” impact reaction to the treatment approach?

CRITIQUE 3

Applicant: Dr. Sheila Frankfurt is an excellent applicant for the CDA2, with excellent training, 21 peer-reviewed publications (most of them relevant for the proposed project), and recipient of a RR&D CDA1 that directly informs the proposed work.

Mentor(s): The mentorship team is superb and well considered for this proposal. The work is sufficiently distinct from those of the mentors and would allow the applicant to develop an independent research career.

Training Program: The training program is excellent and has been responsive to critiques regarding the focus of the training plan.

Scientific Merit:

Significance: The significance of the proposed project is high. There are limited available treatments for moral injury. The Principal Investigator (PI) is taking a painstaking and systematic approach to establish a potential intervention and has clearly deeply considered stages of development and implementation.

Approach: The applicant was very responsive to feedback and has strengthened the approach. The proposal proposes a staged process of developing a treatment manual for an intervention targeting the impact of morally injurious events on functioning and quality of life. There is strong justification provided for this approach, including multiple stages of testing and development, as well as integration of Veteran-centered and expert feedback. The project's focus on integrating models from both implementation science and treatment development is also highly innovative. Overall, the approach is systematic and rigorous.

Resources: The VISN17 CoE and Texas A&M University Health Science Center provide excellent resources for training and research infrastructure.

Protection of Human Subjects: Appears adequate.

Inclusion of Women, Minorities, and Children: Adequate.

Critique of Vertebrate Animals Section:

	Yes	No
Research with vertebrate animals?		X

Biohazards and Radioisotopes: Not applicable.

Additional Review Criteria (unscored): Not applicable.

Budget (unscored)**:** Appears adequate.

Data Management and Access Plan (for data sharing, unscored)**:** Appears adequate.

Overall Strengths: This is an excellent proposal from a promising CDA2 applicant, with a superb mentorship team and training plan. Dr. Frankfurt's prior experience and scientific output to date will provide a great framework for conducting the proposed study. Her mentorship and training plan will help support a pathway to an independent VA research career focusing on treatment of trauma from a rehabilitation perspective. The proposed study has the potential to make a significant contribution to the VA. The study is well designed, with a painstaking, systematic process that is likely to provide effective treatment development.

Overall Weaknesses: Few.

MEETING ROSTER

Career Development Program - Panel I
Rehabilitation Research and Development Parent IRG
Office of Research & Development
RRD8

RRD8
02/22/2021

CHAIRPERSON(S)

NAYLOR, JENNIFER C, PHD
PSYCHOLOGIST
DURHAM VA MEDICAL CENTER
ASSOCIATE PROFESSOR
DEPT OF PSYCHIATRY
DUKE UNIVERSITY MEDICAL CENTER
DURHAM, NC 27705

PADGETT, LYNNE S, PHD
HEALTH PSYCHOLOGIST
DEPARTMENT OF VETERANS AFFAIRS
VETERANS HEALTH ADMINISTRATION
WASHINGTON DC VA MEDICAL CENTER
WASHINGTON, DC 20422

MEMBERS

BALLARD-CROFT, CHERRY, PHD
ASSOCIATE PROFESSOR
SURGERY DEPARTMENT, CARDIOTHORACIC DIVISION
UNIVERSITY OF KENTUCKY
LEXINGTON, KY 40536

BARNES, SEAN, PHD *
CLINICAL/RESEARCH PSYCHOLOGIST
ROCKY MOUNTAIN MIRECC
DENVER VA MEDICAL CENTER
DENVER, CO 80220

BELANGER, STEPHANIE, PHD *
CO SCIENTIFIC DIRECTOR (INTERIM)
CANADIAN INSTITUTE FOR MILITARY AND
VETERAN HEALTH RESEARCH
ASSOCIATE PROFESSOR
ROYAL MILITARY COLLEGE OF CANADA
KINGSTON, ON K7K 7B4
CANADA

BROCK, JOHN H., PHD *
RESEARCH HEALTH SCIENTIST SPECIALIST
VA SAN DIEGO HEALTHCARE SYSTEM
LABORATORY SCIENTIST
CENTER FOR NEURAL REPAIR
UNIVERSITY OF CALIFORNIA, SAN DIEGO
SAN DIEGO, CA 92093

CARBONE, LAURA D, MD *
STAFF PHYSICIAN
CHARLIE NORWOOD VA MEDICAL CENTER
PROFESSOR AND CHIEF
DIVISION OF RHEUMATOLOGY AND ADULT ALLERGY
GEORGIA REGENTS UNIVERSITY
AUGUSTA, GA 30912

DULIN, JENNIFER N, PHD
ASSISTANT PROFESSOR
TEXAS A&M UNIVERSITY
COLLEGE STATION, TX 77843

GULLBRAND, SARAH E, PHD *
RESEARCH HEALTH SCIENTIST
CORPORAL MICHAEL J. CRESCENZ VA MEDICAL CENTER
RESEARCH ASSISTANT PROFESSOR
UNIVERSITY OF PENNSYLVANIA
PHILADELPHIA, PA 19801

HANSEN, ANDREW H., PHD *
RESEARCH BIOMEDICAL ENGINEER
MINNEAPOLIS VA HEALTH CARE SYSTEM
PROFESSOR OF REHABILITATION SCIENCE
AND BIOMEDICAL ENGINEERING
UNIVERSITY OF MINNESOTA
MINNEAPOLIS, MN 55417

HARRIS, JEANETTE IRENE, PHD
CLINICIAN-INVESTIGATOR
BEDFORD VA HEALTH CARE SYSTEM
BEDFORD, MA 01730

HERROLD, AMY, PHD *
RESEARCH HEALTH SCIENTIST
EDWARD HINES JR. VA HOSPITAL
LOYOLA UNIVERSITY MEDICAL CENTER
CHICAGO, IL 60153

HOWLAND, DENA R., PHD *
HEALTH SCIENTIST
ROBLEY REX VA MEDICAL CENTER
ASSOC PROFESSOR, DEPT OF NEUROLOGICAL SURGERY
UNIVERSITY OF LOUISVILLE
KY SPINAL CORD INJURY RESEARCH CTR
LOUISVILLE, KY 40292

LEE, SANG WOOK, PHD *
ASSISTANT PROFESSOR
DEPARTMENT OF BIOMEDICAL ENGINEERING
CATHOLIC UNIVERSITY OF AMERICA
WASHINGTON, DC 20064

MEEK, SANFORD GEORGE, PHD *
ASSOCIATE PROFESSOR
UNIVERSITY OF UTAH
DEPARTMENT OF MECHANICAL ENGINEERING
SALT LAKE CITY, UT 84112

NIEUWSMA, JASON A, PHD *
PSYCHOLOGIST, MENTAL HEALTH
DURHAM VA MEDICAL CENTER
DEPARTMENT OF PSYCHOLOGY
UNIVERSITY OF WYOMING
LARAMIE, WY 82071

PEARSE, DAMIEN D., PHD *
RESEARCH HEALTH SCIENTIST
MIAMI VA MEDICAL CENTER
PROFESSOR
UNIVERSITY OF MIAMI MILLER SCHOOL OF MEDICINE
MIAMI, FL 33136

PLESS KAISER, ANICA, PHD *
CLINICAL RESEARCH PSYCHOLOGIST
VA BOSTON HEALTHCARE SYSTEM
NATIONAL CENTER FOR PTSD
RESEARCH ASSISTANT PROFESSOR, PSYCHIATRY DEPART
BOSTON UNIVERSITY SCHOOL OF MEDICINE
BOSTON, MA 02130

RASETSHWANE, DANIEL M., PHD
SCIENTIST
BOYSTOWN NATIONAL RESEARCH HOSPITAL
OMAHA, NE 68131

SCHEIBEL, RANDALL S., PHD *
RESEARCH HEALTH SCIENTIST
MICHAEL E. DEBAKEY VA MEDICAL CENTER
COGNITIVE NEUROSCIENCE LABORATORY
PROFESSOR, PHYSICAL MEDICINE AND REHAB
BAYLOR COLLEGE OF MEDICINE
HOUSTON, TX 77030

SCOTT, JAMES COBB, PHD *
CLINICAL PSYCHOLOGIST
PHILADELPHIA VA MEDICAL CENTER
ASSISTANT PROFESSOR
UNIVERSITY OF PENNSYLVANIA
PHILADELPHIA, PA 19072

SKELTON, FELICIA, MD
STAFF PHYSICIAN
MICHAEL E DEBAKEY VA MEDICAL CENTER
ASSISTANT PROFESSOR
PHYSICAL MEDICINE & REHABILITATION
BAYLOR COLLEGE OF MEDICINE
HOUSTON, TX 77030

SOWERS, LEVI P, PHD
RESEARCH HEALTH SCIENCE SPECIALIST
IOWA CITY VA HEALTH CARE SYSTEM
RESEARCH SCIENTIST
DEPARTMENT OF MOLECULAR PHYSIOLOGY AND BIOPHYSICS
UNIVERSITY OF IOWA CARVER COLLEGE OF MEDICINE
IOWA CITY, IA 52242

YAMAGUCHI, DEAN T, MD
EMERITUS PROFESSOR OF MEDICINE
UNIVERSITY OF CALIFORNIA, LOS ANGELES
LOS ANGELES, CA 90095

YOON, JONG H, MD *
PHYSICIAN
VA PALO ALTO HEALTH CARE SYSTEM
ASSISTANT PROFESSOR
PSYCHIATRY AND BEHAVIORAL SCIENCES
STANFORD UNIVERSITY
STANFORD, CA 94305

EXECUTIVE SECRETARY

BOUWER, H. G. ARCHIE, PHD
DEPUTY ASSOCIATE CHIEF OF STAFF
PORTLAND VA HEALTH CARE SYSTEM
SOM-MOLECULAR MICROBIOLOGY & IMMUNOLOGY DEPT
OREGON HEALTH SCIENCES UNIVERSITY
PORTLAND, OR 97213

SCIENTIFIC REVIEW OFFICER

GROER, SHIRLEY, PHD
SCIENTIFIC PROGRAM MANAGER
DEPARTMENT OF VETERANS AFFAIRS
OFFICE OF RESEARCH AND DEVELOPMENT
REHABILITATION RESEARCH AND DEVELOPMENT SERVICE
WASHINGTON, DC 20420

* Temporary Member. For grant applications, temporary members may participate in the entire meeting or may review only selected applications as needed.

Consultants are required to absent themselves from the room during the review of any application if their presence would constitute or appear to constitute a conflict of interest.
